# Processing of Complement Coercion With Aspectual Verbs in Mandarin Chinese: Evidence From a Self-Paced Reading Study

**DOI:** 10.3389/fpsyg.2021.643571

**Published:** 2021-05-31

**Authors:** Wenting Xue, Meichun Liu, Stephen Politzer-Ahles

**Affiliations:** ^1^Department of Linguistics and Translation, City University of Hong Kong, Hong Kong, China; ^2^Department of Chinese and Bilingual Studies, The Hong Kong Polytechnic University, Hong Kong, China

**Keywords:** complement coercion, type mismatch, semantic enrichment, self-paced reading, Mandarin Chinese, aspectual verbs

## Abstract

This study examines whether Chinese complement coercion sentences with aspectual verbs will elicit processing difficulty during real-time comprehension. *Complement coercion* is a linguistic phenomenon in which certain verbs (e.g., *start, enjoy*), requiring an event-denoting complement, are combined with an entity-denoting complement (e.g., *book*), as in *The author started a book*. Previous studies have reported that the entity-denoting complement elicited processing difficulty following verbs that require event argument compared with verbs that do not (e.g., *The author wrote a book*). While the processing of complement coercion has been extensively studied in Indo-European languages such as English and German, it is relatively under-researched in Sino-Tibetan languages such as Mandarin Chinese. Given the fact that there are many linguistic elements behaving distinctly in the different language families, for instance, verbs with respect to their semantic properties and syntactic representations of the complement, it is meaningful to investigate whether or not the existing linguistic differences have any effect on the processing of complement coercion in Mandarin. With this research goal, we recorded self-paced reading time of 61 native Mandarin speakers to investigate the processing of the entity-denoting complement in sentences with three different verb types (*aspectual verbs* which require an event-denoting complement, *preferred verbs* which denote a preferred interpretation of the aspectual expressions, and *non-preferred verbs* which denote a non-preferred but plausible interpretation of the aspectual expressions), as exemplified in 顾客开始/填写/查看这份问卷 *gù-kè kāi-shǐ/tián-xiě/chá-kàn zhè-fèn wèn-juàn* “The customer started/filled in/checked the questionnaire.” It was found that the entity noun complement (e.g., 这份问卷 *zhè-fèn wèn-juàn* “the questionnaire”) elicited significantly longer reading times in coercion sentences than non-coercion counterparts. The results are compatible with the previous findings in English that complement coercion sentences impose processing cost during real-time comprehension. The study contributes empirical evidence to coercion studies cross-linguistically.

## Introduction

Sentence comprehension is an intriguing and complex system in which comprehenders often have to go beyond what is explicitly expressed to infer additional information. This process may sometimes incur processing cost.

One linguistic phenomenon involving information not explicitly expressed is complement coercion, which involves repairing a semantic type mismatch between an event-selecting verb and an entity-denoting complement (Pustejovsky, 1991, 1995; Jackendoff, 1997). This phenomenon is found in sentences such as (1) below. The verb *start* appears to be an event-selecting verb, which normally takes an eventive complement, as *started reading the novel* in (1a). But this verb can also co-occur with a nominal complement denoting an entity, as *started the novel* in (1b). What is noticeable in (1b) is that there is a type clash between the verb and its nominal complement, which does not overtly indicate what activity Mary started doing. To remedy the clash, the noun complement is supposed to undergo a type shift from an entity to an event (i.e., changing the meaning of *the novel* to something like “the process of reading the novel”) so that it meets the selectional restriction of the verb.


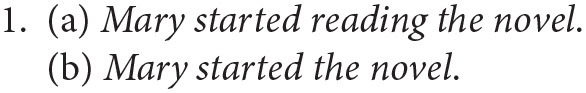


Expressions requiring complement coercion have been reported to trigger processing difficulty compared to those no need of complement coercion (e.g., McElree et al., 2001; Traxler et al., 2002; Pickering et al., 2005; Frisson and McElree, 2008; Kuperberg et al., 2010; Husband et al., 2011; Lai, 2017; Spalek and Tomaszewicz, 2018). This topic has been well-studied in Indo-European languages such as English, German, but is relatively under-researched in Sino-Tibetan languages such as Mandarin Chinese. Therefore, the current study will examine the processing of complement coercion in Mandarin Chinese with a self-paced reading paradigm, focusing on aspectual verbs which are acknowledged as typical event-selecting verbs, and according to some studies (e.g., Katsika et al., 2012), the most prominent coercing verbs.

## Processing Difficulty of Complement Coercion Reported in Previous Studies

A number of psycho/neurolinguistic studies have reported that processing expressions that require complement coercion is more taxing than processing non-coercion controls (e.g., McElree et al., 2001, 2006; Piñango and Zurif, 2001; Traxler et al., 2002, 2005; Pickering et al., 2005; Pylkkänen and McElree, 2007; Frisson and McElree, 2008; Baggio et al., 2010; Kuperberg et al., 2010; Husband et al., 2011; Katsika et al., 2012; Husband and Politzer-Ahles, 2016; Delogu et al., 2017; Lai, 2017; Zarcone et al., 2017; Spalek and Tomaszewicz, 2018). For example, McElree et al. (2001) had participants read sentences such as *The secretary ____ the memo before the annual sales conference*, with one of three types of verbs preceding the critical noun phrase *the memo*: (a) Coercing verbs (e.g., *begin, enjoy*), which semantically select for an event-denoting complement; (b) Preferred verbs (e.g., *type*), which trigger preferred interpretation for coercion sentences; (c) Non-preferred verbs (e.g., *read*), which trigger non-preferred but plausible interpretation for coercion sentences. It was found that *the memo* is slower to process in the coercion context than the other contexts. In their event-related potentials study, Kuperberg et al. (2010) found an N400 effect for coerced complement nouns compared with non-coerced ones during the real-time comprehension.

Three different explanations have been proposed to account for why objects that trigger coercion elicit more processing demands relative to objects that don't. One explanation is that the processing cost is related to semantic processes particular to these sorts of coercion-eliciting sentences (i.e., the reading time slowdown reflects the additional time taken to change the semantic type of the object, or, more likely, to recover from noticing the type mismatch between the verb and the object) (e.g., McElree et al., 2001; Traxler et al., 2002, 2005; Frisson and McElree, 2008; Pylkkänen et al., 2009; Baggio et al., 2010; Kuperberg et al., 2010; Spalek and Tomaszewicz, 2018). These processes lead to an enriched form of semantic composition (Jackendoff, 1997). The alternative account, offered by *surprisal* theory (Hale, 2001; Levy, 2008), maintains that the processing cost results more likely from the relatively lower predictability (thus, high surprisal) of the entity-denoting object preceded by an event-taking verb (Delogu et al., 2017). Under this account, the processing cost observed is not specific to complement coercion, but is just a reflection of what always happens when comprehenders encounter less-expected input. Another account, resorting to lexical functions encoded in the coercing verbs, addresses that the coercing verbs lexically encode a set of dimensions (e.g., spatial, temporal, eventive, informational). The multiple dimensions are selected in a given context to have one mapping on the denotation of the object (Piñango and Deo, 2016). The account suggests that reading time slowdowns of complement coercion are more likely yielded from the ambiguity of multiple dimension readings and the selection of the exact dimension mapped onto the denotation of the object argument, rather than the type-shifting operations to repair the type mismatch (Lai, 2017). The issue of whether these reading time slowdowns reflect specific semantic operations or domain-general surprisal is outside of the scope of the current study. The main purpose of the present study is to examine whether Chinese sentence comprehension also shows this processing phenomenon, regardless of what its locus is; this can pave the way to further investigate the locus of the effect in future follow-up studies.

## Complement Coercion in Mandarin Chinese

As mentioned above, the processing of complement coercion has been studied extensively in Indo-European languages (e.g., English, German). While there has little psycholinguistic research on complement coercion in Mandarin, there have been several corpus-based and metalinguistic examinations of the phenomenon (Lin and Liu, 2005; Liu, 2005; Lin et al., 2009; Hsu and Hsieh, 2013; Song, 2015; Xue et al., 2020).

Lin and Liu (2005) claimed that complement coercion does not occur in Mandarin. They found that a Mandarin example sentence 张三开始一本书 *Zhāng-sān kāi-shǐ y*&#*x00131;̄-běn shū*, directly translated from the English complement coercion sentence *John began a book*, is judged as unnatural by native Mandarin speakers, even though the English sentence is acceptable to native English speakers. They argued that the Mandarin version is only acceptable if a verb is inserted between the matrix verb 开始 *kāi-shǐ* “start/begin” and the noun phrase ⇀本书 *y*&#*x00131;̄-běn shū* “a book” to explicitly express an event associated with the complement referent, e.g., 读 *dú* “read” or 写 *xiě* “write”. Their claims are based on introspection, however, rather than on surveys, and it is likely that their observations about Mandarin would also apply to English (i.e., most English speakers would also find *John began reading a book* more acceptable than *John began a book*); this challenges the conclusion that Mandarin is fundamentally different from English.

Their claim that Mandarin does not have complement coercion has also been challenged by subsequent studies providing evidence for complement coercion in Mandarin (Liu, 2005; Lin et al., 2009; Hsu and Hsieh, 2013; Song, 2015; Xue et al., 2020). Liu (2005) presented a case study of the transitive use of the manner-denoting verb 赶 *gǎn* “rush” in the construction (赶 *Gan* + Noun) from the perspective of Construction Grammar (Goldberg, 1995). She found that the meaning of the construction cannot be directly retrieved from either the verb or the noun but has to be constructionally coerced into the proper interpretation. For instance, 赶报告 *gǎn bào-gào* “rush (to finish) a paper” requires a semantic inference from the noun's denotation to retrieve the under-specified activity, i.e., “writing the paper”. Subsequently, Lin et al. (2009) and Hsu and Hsieh (2013) demonstrated that these constructions occur in natural corpora. Most recently, Xue et al. (2020) conducted an acceptability task to ask native Mandarin speakers to judge the acceptability of Mandarin sentences with complement coercion construction. The results showed that the coercion sentences, similar to non-coercion counterparts, are acceptable to native speakers, but just with relatively lower ratings. The linguistic phenomenon is also elaborated by Song (2015) from the perspective of Generative Lexicon (Pustejovsky, 1995). But Song also claimed that the linguistic phenomenon is less pervasive in Mandarin compared with English, because some Mandarin equivalents of the English coercing verbs (e.g., 试图/企图 *shì-tú*/*qǐ-tú* “attempt”) are not available in the coercion construction in Mandarin. But the presence of verbs that can coerce objects in English while their Mandarin translation equivalents cannot do so is not proof that coercion is less frequent in Mandarin than in English, because there are also converse cases (verbs that can coerce their complements in Mandarin, such as 赶 *gǎn* “rush to finish”, but whose English translation equivalents cannot do so). In addition, Song (2015, p. 150–151) claimed that some of the Mandarin equivalents of English coercing verbs, such as 开始 *kāi-shǐ* “begin/start”, cannot combine with a complement denoting an entity, and have weaker/little potential for coercion.

If the above-mentioned two claims in Song's study are true, i.e., (1) complement coercion is less pervasive in Mandarin compared to in English, and (2) Mandarin event-selecting verbs trigger weaker/little coercion to the entity complement, then it is quite possible that Mandarin sentences with complement coercion structure may not elicit processing cost.

Although complement coercion in Mandarin has drawn attention of Chinese linguists, little empirical evidence has been provided to figure out the underlying processing mechanism of this sentence structure. To our knowledge, only Xue and Liu (2020) conducted a pilot study of self-paced reading to examine the processing profile of the entity-denoting noun complement preceded by a wide range of event-selecting verbs (e.g., aspectual verbs, psychological verbs, other verbs etc.). Their findings showed that significantly longer reading time was not found on the entity noun of coercion sentences where the type mismatch occurs; instead, it was found on the word directly after the noun. The result reflects a typical spill-over effect; but it is also probably due to the inclusion of semantically heterogeneous stimuli with various verb types in coercion condition, as pointed out by the authors themselves. Therefore, it is necessary to refine the study by examining the coercion processing with well-controlled stimuli, saying, only with aspectual verbs whose meaning introduces the initiation, termination, or continuation of an activity (Levin, 1993); they have been acknowledged to be typical event-selecting coercing verbs, and have been argued by Song (2015) to have weaker/little potential for coercion in Mandarin.

The current self-paced reading experiment was designed to investigate whether or not the entity noun would exhibit reading time slowdowns when preceded by aspectual verbs requiring an eventive complement, compared to when preceded by verbs not requiring an eventive complement. We predicted that although there are differences in terms of specific characteristics of lexical semantics between Mandarin and English event-selecting verbs, as pointed out in Song (2015), the fundamental processing mechanism of complement coercion may be identical; thus, the Mandarin expressions with aspectual verbs would yield longer reading time than those without event-selecting verbs.

## Methods

This study was carried out in accordance with the Declaration of Hong Kong, and was approved by the Ethics Committee of the Department of Linguistics and Translation, City University of Hong Kong.

### Participants

Sixty-one native Mandarin speakers (37 women and 24 men; age: *mean* = 25 years; *range* = 19–30) from City University of Hong Kong were recruited for the current experiment. All participants had normal or corrected to normal vision, and none of them reported any language disorder. They all gave their written informed consent to the participation, and received a monetary reward after completing the experiment.

### Materials

Thirty-two triplets of experimental stimuli were generated for the self-paced reading experiment, all with 14–16 characters. An example of the materials is presented in Table 1 [see Supplementary Material for all items, in which the abbreviations involved are cited from Li and Thompson (1981)]. Following the paradigm of Traxler et al. (2002) (Exp. 1), three verb types were manipulated to take an entity-denoting noun complement: (a) Aspectual verbs (coercion), which semantically select an event-denoting complement; (b) Preferred verbs (non-coercion), which denote a preferred interpretation to the coercion expressions; (c) Non-preferred verbs (non-coercion), which denote a non-preferred but plausible interpretation to the coercion expressions.

**Table 1:** A samples set of stimuli.

**Verb Type**		**Verb**	**NP**	**NP + 1**	**NP + 2**	
Aspectual verbs	顾客 *gù-kè* customer	开始 *kāi-shǐ* start	这份问卷 *zhè-fèn wèn-juàn* this-CL questionnaire	之前 *zhı̄-qián* before	看过 *kàn-guò* take a look	身份证 *shēn-fèn zhèng* ID card
	“The customer took a look of the ID card before starting the questionnaire.”					
Preferred verbs	顾客 *gù-kè* customer	填写 *tián-xiě* fill in	这份问卷 *zhè-fèn wèn-juàn* this-CL questionnaire	之前 *zhı̄-qián* before	看过 *kàn-guò* take a look	身份证 *shēn-fèn zhèng* ID card
	“The customer took a look of the ID card before filling in the questionnaire.”					
Non-preferred verbs	顾客 *gù-kè* customer	查看 *chá-kàn* check	这份问卷 *zhè-fèn wèn-juàn* this-CL questionnaire	之前 *zhı̄-qián* before	看过 *kàn-guò* take a look	身份证 *shēn-fèn zhèng* ID card
	“The customer took a look of the ID card before checking the questionnaire.”					

*CL, classifier. The complement NP is represented with a composition of (demonstrative + CL + noun_entity−type_) to denote concrete or specific entities. There are different types of classifiers in Mandarin, one of which is event classifiers. These classifiers select process nouns (Huang et al., 1995) and can coerce a nominal to express an event (Huang and Ahrens, 2003). In the current study, we excluded 35 event classifiers listed in Huang et al. (1995) to ensure that there was no event reading encoded in the noun complement*.

Three norming tests were carried out to select the stimuli: *preference norming* to derive preferred and non-preferred verbs, *acceptability norming* to rate the acceptability of the stimuli, as well as *cloze norming* to assess the predictability of the critical complement noun where the type mismatch appears. Before the norming tests, we first made 55 coercion expressions composed with a subject, an event-selecting verb, and an entity-denoting object, like 顾客开始这份问卷 *gù-kè kāi-shǐ zhè-fèn wèn-juàn* “The customer started the questionnaire”.

The subject of the sentences was created by using more informative subjects (Traxler et al., 2002) rather than proper names (Delogu et al., 2017), such as 顾客 *gù-kè* “customer”, 医生 *y*&#*x00131;̄-shēng* “doctor”, to facilitate the selection of the preferred verbs for the coercion strings in the *preference norming* test (see details in the following).

The object of the sentences represents an entity rather than an event/activity. They all are composed of an entity noun modified by a demonstrative 这/那 *zhè/nà* “this/that” together with an entity-type classifier (e.g., 份 *fèn* is used for something like newspapers, reports etc., 本 *běn* for something like books, 栋 *dòng* for something like buildings etc.).

The event-selecting verbs in this study are five aspectual verbs: 开始 *kāi-shǐ* “begin/start”, 继续 *jì-xù* “continue”, 完成 *wán-chéng* “finish”, 结束 *jié-shù* “end”, 停止 *t*í*ng-zhǐ* “stop”. They were selected based on linguistic diagnostics for raising verbs. According to Rochette (1999), all usages of aspectual verbs can be considered as raising predicates which do not impose selectional restrictions on their surface subject; the subject is restricted by the embedded predicate instead. In the coercion sentence顾客开始这份问卷 *gù-kè kāi-shǐ zhè-fèn wèn-juàn* “The customer started the questionnaire”, the surface subject 顾客 *gù-kè* “customer” can be also interpreted as the subject of the implicit predicate, e.g., 填写 *tián-xiě* “fill in”, such that the sentence has the meaning like “it is the customer who filled in the questionnaire”, but not “it is someone else who filled in the questionnaire”. This can be exemplified in (2a) in which the other participant “the volunteer” is involved, but the sentence is ungrammatical. Further, aspectual verbs do not introduce an independent event; rather, they function as “aspectual modifiers” which modify the event expressed by the embedded predicate and its argument (Rochette, 1999). The coercion sentence does not involve two events described by *start* and *filling in the questionnaire* but rather introduces only one single event of *start to fill in*. This can be tested in sentences like (2b) in which two distinct time reference adverbials (i.e., 早上 *zǎo-shàng* “in the morning” and 下午*xià-wǔ* “in the afternoon”) are included, but the sentence is ungrammatical. All the five aspectual verbs have the similar usages (Cao, 1996), and thus selected as the target verbs for the present study.


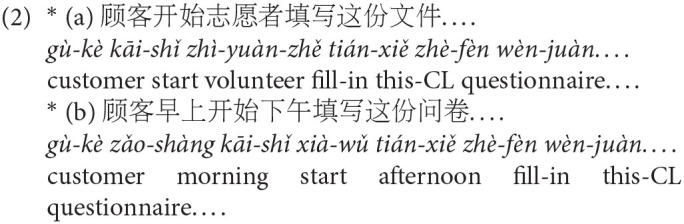


#### Preference Norming

Twenty-five native Mandarin speakers were invited to provide two-character verbs in 55 coercion strings like 顾客开始___这份问卷 *gù-kè kāi-shǐ*__*zhè-fèn wèn-juàn* “The customer started__the questionnaire.” Two examples were given at the beginning of the test, e.g., 小雅阅读这份报纸 *xiǎo-yǎ* *yuè-dú* *zhè-fèn bào-zhǐ* “Ms. Ya read the newspaper.” Thirty-two test sentences were selected such that the dominant responses occurred six (24%) or more times among the participants' responses. The verbs selected for the preferred condition (e.g., 填写 *tián-xiě* “fill in”) were those occurred most frequently, 11.50 times (out of 25) on average, ranging from 6 to 23 times; the verbs for the non-preferred condition (e.g., 查看 *chá-kàn* “check”) were those occurred least frequently, 1.50 times on average, ranging from 1 to 5 times. We did not use verbs that never occurred for non-preferred candidates to try to minimize the possibility of making cloze probability of the complement noun very low. After the norming test, 32 triplets of expressions were determined.

#### Acceptability Norming

To assess the acceptability of the 32 triplets of experimental stimuli, sixty native Mandarin speakers, who did not participate in the preference norming test, were invited to rate the acceptability on a scale of 6 (1 = completely unacceptable, 2 = almost unacceptable, 3 = marginally unacceptable, 4 = marginally acceptable, 5 = almost acceptable, and 6 = completely acceptable) based on their intuition. We used a scale with an even number of levels to avoid over-selection of the midpoints (Weems and Onwuegbuzie, 2001). The experimental stimuli were randomly distributed to three lists with a Latin Square design. This was to make sure that none of the three conditions of each item occurred in the same list. Note, however, that since the 32 items could not be separated to the three lists equally, each list contained two conditions with 11 items and another condition with 10 items. Besides 32 experimental sentences in each list, 32 filler sentences were also made with 14–16 characters, same as the character numbers of the experimental sentences. The three lists were distributed to the sixty participants at random, each with twenty. No data were excluded in the acceptability norming test. Mean acceptability ratings for the aspectual verb sentences, preferred verb sentences, and non-preferred verb sentences were 4.06, 4.77, and 4.52, respectively. All means were higher than 3.5 (the midpoint of the scale), which indicated that all three types of sentences were acceptable to Mandarin speakers in general.

#### Cloze Norming

Due to the semantic properties of the three conditions manipulated, it is inevitable that the predictability of the complement noun, following the three types of verbs, must be distinct, which may consequently engender discrepancy in the processing patter of the noun together with any effect incurred by the condition/verb type. It is necessary, therefore, to evaluate the target noun's predictability in the three sentence types. Sixty native Mandarin speakers, who did not participate in either the preference norming test or the acceptability norming test, were asked to take the cloze norming with the 32 triplets generated in the preference norming. The participants were asked to provide three-character noun phrases to strings up to and including a demonstrative这/那 *zhè / nà* “this/that”, like 顾客开始/填写/查看这___ *gù-kè kāi-shǐ/ tián-xiě/chá-kàn zhè*___ “The customer started/filled-in/checked this___.” Two examples were shown to the participants in the instruction part of the test, such as 记者报道这则新闻 *jì-zhě bào-dào zhè-zé xin-wén* “The journalist reported the news.” The 32 triplets of test sentences were randomly distributed to three lists with a Latin Square design so that none of the three conditions of each item occurred in the same list. Similar to the acceptability norming test, each list contained two conditions with 11 items and another condition with 10 items. The three lists were randomly distributed to the sixty participants, each with twenty. No participants' responses were removed.

Participants' responses were compared with the target words (i.e., the complement noun) of the test sentences. The responses were counted as the same as the target words based on two criteria: (1) the noun of the completed strings is the same as the noun of the complement of test sentences; (2) the classifier is not an event type in that an event classifier can coerce its modified entity-type noun an event reading (Huang and Ahrens, 2003). In terms of the first criterion, for example, responses like 这部小说 *zhè-bù xiǎo-shuō* would be counted as the same as the target word 这本小说 *zhè-běn xiǎo-shuō* “this novel” in that the noun of the two phrases is the same 小说 *xiǎo-shuō* “novel”, even though the classifiers are different 部 *bù* vs. 本 *běn*). In terms of the second criterion, responses like 这场电影 *zhè-chǎng diàn-yǐng* would be not counted as the same as the target word 这部电影 *zhè- bù diàn-yǐng* “this movie”. This is because the classifier 场 *chǎng* is an event classifier, referring to “scheduled and regularly occurring events” (Huang and Ahrens, 2003, p. 6); thus, the composition of the complement noun phrase 这场电影 *zhè-chǎng diàn-ying* may express an event, which was not a qualified entity-denoting complement in this study. Cloze probabilities of the complement noun (proportion of the participants' responses completed with the target words) (Traxler et al., 2002, p. 534) following the aspectual verbs, preferred verbs, and non-preferred verbs were 0.06 (*range* = 0–0.45), 0.39 (*range* = 0.05–0.90), and 0.15 (*range* = 0–0.40), respectively.

The experimental stimuli used in the self-paced reading experiment were adapted from the 32 triplets of sentences in the acceptability norming test. They were randomly distributed to three lists with Latin Square design, and only one version of each item occurred in each list. Thirty-two filler sentences with various sentence structures were also inserted to avoid the participants' awareness of the purpose of the research. To help participants familiarize the procedures, six items were practiced at the beginning of the list. Thus, 70 sentences in total were included. Note that this group of participants did not attend any of the three norming tests mentioned above.

### Procedures

The experiment was conducted in a quiet study room at the library of City University of Hong Kong. Participants were assigned with one of the three lists randomly and instructed to read sentences at their own pace. The experiment began with the presentation of written instructions on the screen, which were verbally reinforced by the experimenter. Before the real trials, a practice session was carried out to familiarize the participants with the experiment procedure. The experimental session lasted for approximately 15 minutes.

The sentences were presented segment-by-segment (as shown in Table 1) with a moving window procedure, with white characters (font Kai 14) on a black background. This was achieved through DMDX software (Forster and Forster, 2003). Each trial began with a cross sign “+”, and the first segment appeared upon pressing space bar. With the subsequent press, the following segment appeared, and the previous segment was replaced by a set of dashes. When finishing the reading of a sentence, participants were asked to answer a yes-or-no comprehension question relevant to the content of the sentences to ensure that they paid attention to the task. They responded by pressing Yes or No button on the keyboard. As long as the response was given, the next trial started. All sentences were presented randomly. The computer recorded the participants' responses to each question and reading time to each segment from the time a segment first appeared until the subsequent press of the space bar.

## Data Analysis

Statistical analyses were conducted in reading times (RTs) of four regions: the region containing the verb (e.g., 开始/填写/查看 *kāi-shǐ*/ *tián-xiě*/*chá-kàn* “start/fill in/check”), the critical region containing the complement noun phrase (NP, e.g., 这份问卷 *zhè-fèn wèn-juàn* “the questionnaire”), the post-critical region (NP + 1, e.g., 之前 *zhi-qián* “before”), and the subsequent region (NP + 2, e.g., 看过 *kàn-guò* “take a look”). The NP region was what we were focused on, to examine the potential processing cost; the verb region was examined to see whether there was any processing difference observed at the verbs, which might further influence the processing of the critical NP region; the NP + 1 and NP + 2 were two regions where spillover effects (if took place) might be observed.

Prior to the analysis, data were cleaned according to two separate measures (Dempsey et al., 2020). First, participants were excluded based on how accuracy scores to all comprehension questions (<75%) which were calculated in Excel. After this was completed, one participant was removed for low accuracy. Second, reading times for words in the above-mentioned four regions of critical trials were excluded from analysis if they were longer than 2,000 ms or shorter than 100 ms. This results in a loss of 23 reading time data points, corresponding to 0.30% of reading time data.

**Figure 1:**
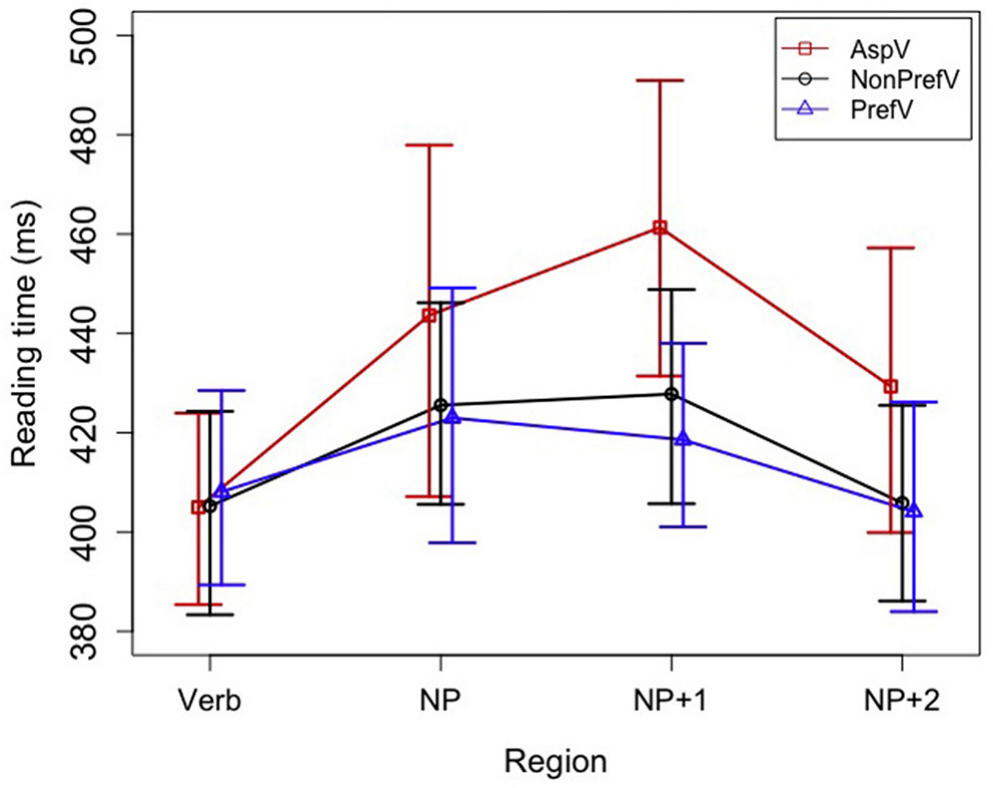
Mean reading times by verb type and region. The error bars indicate the difference-adjusted 95% (percentile) mixed-effect-model-based intervals (Politzer-Ahles, 2017). The intervals can be generally interpreted as indicating that when one sentence type's interval does not include another sentence type's mean, the two types are likely (but not guaranteed) to be significantly different in a mixed effect model.

The rest of data were analyzed in the R statistical environment (R Core Team, 2018). To get an overview of reading times for each type of sentences at each region, mean reading times [using the tapply() function of the plyr package (Wickham, 2011) in R] and difference-adjusted 95% (percentile) mixed-effect-model-based intervals (Politzer-Ahles, 2017) were first calculated. The intervals indicate roughly that when one sentence type's interval does not include another sentence type's mean, the two types are likely (but not guaranteed) to be significantly different in a mixed effect model (Politzer-Ahles, 2017; Politzer-Ahles and Piccinini, 2018). Results are reported in Table 2 and illustrated in Figure 1.

**Table 2:** Mean reading times (in milliseconds) at each region, and difference-adjusted 95% (percentile) mixed-effect-model-based intervals (Politzer-Ahles, 2017).

**Verb type**	**Verb**	**NP**	**NP + 1**	**NP + 2**
Aspectual verbs	404.99 (387.20, 423.53)	443.64 (406.76, 480.12)	461.33 (431.65, 488.76)	429.33 (400.67, 457.12)
Preferred verbs	408.08 (389.97, 428.00)	422.98 (398.45, 448.09)	418.59 (400.74, 437.81)	404.10 (384.89, 425.53)
Non-preferred verbs	405.22 (383.94, 425.06)	425.54 (405.24, 446.95)	427.78 (406.61, 448.66)	405.83 (386.52, 424.88)

Statistical analyses were conducted by performing separate linear mixed-effects models (*lmer*) with the *lme4* package (Bates et al., 2015) on the data from each of the four critical regions. As the dependent variable, the reading times were log-transformed to yield a model with approximately normal residuals. For each region, a linear mixed model was constructed, incorporating the categorical fixed effect of Verb Type (three levels: Aspectual verbs, Preferred verbs, Non-preferred verbs), the continuous fixed effect of NP Predictability, and their interaction. The Verb Type was dummy coded. We started with the full structure of random effects supported by the design, which included crossed random intercepts for both participants and items, and random slope parameters for the main effects of Verb Type, NP Predictability, as well as their interaction. We then simplified the full random effects structure via model comparisons to obtain the maximal fitting model for the data, using α = 0.2 (Matuschek et al., 2017). The main effects of Verb Type ^*^ NP Predictability, Verb Type, and NP Predictability were evaluated by means of likelihood ratio tests (i.e., comparing a model with one factor to the same model but without the factor).^1^ All models are presented in Table 3, and the results of likelihood ratio tests are reported in Table 4. As for the main effect of Verb Type, pairwise comparisons were conducted by summarizing the maximal model [with the summary() function], and then releveling the baseline condition. Results can be found in Table 5.

**Table 3:** Model comparisons: likelihood ratio tests.

**Model**	**Formula**
Full model	RT ~ VT * NPP + (1 + VT * NPP | Subject) + (1 + VT * NPP | Item)
Maximal model	RT ~ VT * NPP + (1 + VT | Subject) + (1 + NPP | Item)
Model 1	RT ~ VT + NPP + (1 + VT | Subject) + (1 + NPP | Item)
Model 2	RT ~ NPP + (1 + VT | Subject) + (1 + NPP | Item)
Model 3	RT ~ VT + (1 + VT | Subject) + (1 + NPP | Item)

*RT, Reading Time; VT, Verb Type; NPP, NP Predictability*.

The interaction of VT ^*^ NPP was evaluated by comparing Maximal model with Model 1.

*The main effect of VT was evaluated by comparing Model 1 with Model 2*.

*The main effect of NPP was evaluated by comparing Model 1 with Model 3*.

**Table 4:** Results of likelihood ratio tests.

**Region**	VT^*^ **NPP**		**VT**		**NPP**	
	**χ^**2**^ (*df*)**	***p*-value**	**χ^**2**^ (*df*)**	***p*-value**	**χ^**2**^ (*df*)**	***p*-value**
Verb	1.350 (2)	0.509	0.401 (2)	0.818	0.071 (1)	0.79
NP	4.997 (2)	0.082	0.093 (2)	0.954	0.044 (1)	0.834
NP + 1	10.54 (2)	0.005^**^	5.276 (2)	0.071.	0.072 (1)	0.788
NP + 2	5.881 (2)	0.053.	7.211 (2)	0.027^*^	0.002 (1)	0.968

*VT, Verb Type; NPP, NP Predictability*.

*** *p ≤ 0.001*;

** *p ≤ 0.01*;

* *p ≤ 0.05*;

*‘.'p ≤ 0.1*.

**Table 5:** Results of pairwise comparisons for the main effect of verb type.

	**Comparison**	***Estimate***	***Std. error***	***df***	***t***	***p***
Verb	AspV vs. PrefV	0.034	0.032	399.302	1.055	0.292
	AspV vs. NonPrefV	0.012	0.026	509.758	0.459	0.646
	PrefV vs. NonPrefV	−0.022	0.035	236.666	−0.622	0.534
NP	AspV vs. PrefV	−0.050	0.037	815.106	−1.377	0.169
	AspV vs. NonPrefV	0.015	0.030	482.638	0.495	0.621
	PrefV vs. NonPrefV	0.065	0.038	1185.904	1.700	0.089.
NP + 1	AspV vs. PrefV	−0.125	0.035	481.393	−3.560	<0.001^***^
	AspV vs. NonPrefV	−0.057	0.029	566.083	−1.963	0.050.
	PrefV vs. NonPrefV	0.069	0.037	539.865	1.856	0.064.
NP + 2	AspV vs. PrefV	−0.095	0.031	1496.282	−3.030	0.002^*^
	AspV vs. NonPrefV	−0.052	0.027	958.687	−1.955	0.051.
	PrefV vs. NonPrefV	0.043	0.034	1521.303	1.285	0.199

*AspV, Aspectual Verb; PrefV, Preferred Verb; NonPrefV, Nonpreferred Verb*.

*** *p ≤ 0.001*;

** *p ≤ 0.01*;

* *p ≤ 0.05*;

*‘.'p ≤ 0.1*.

**Figure 2:**
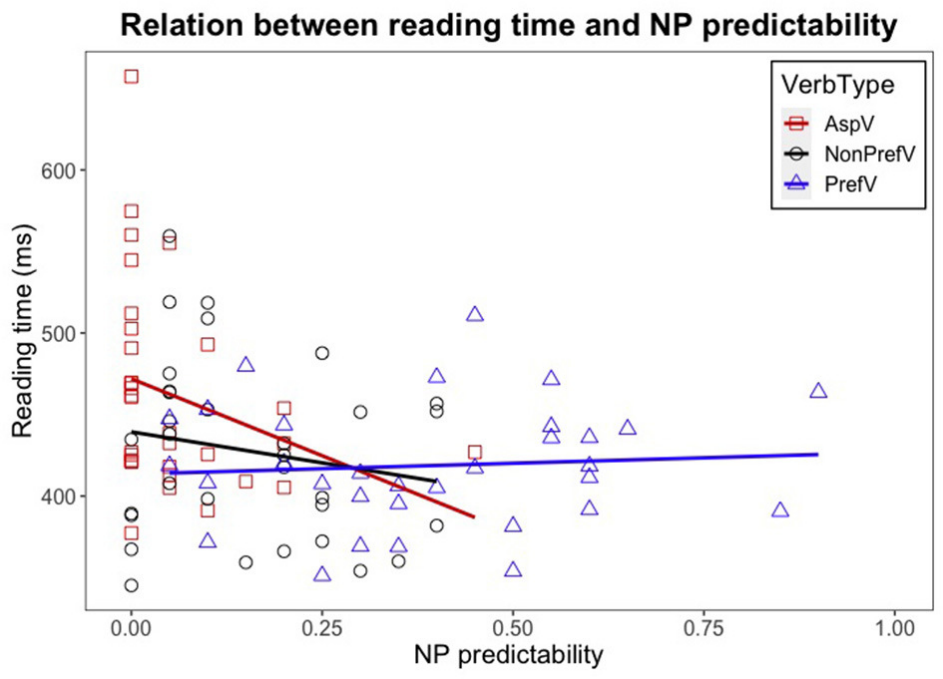
Relation between reading time (at NP + 1) and NP predictability by verb type. The x-axis shows NP predictability, and the y-axis shows reading times (in milliseconds). The red squares represent reading times of NP + 1 for each item in the aspectual verb condition; the black dots represent reading times of NP + 1 in the non-preferred verb condition; and the blue triangles represent the reading times of NP + 1 in the preferred verb condition. The three lines on this scatter plot indicate linear regression lines for the three verb types, the red line for the aspectual verb condition, the black line for the non-preferred verb condition, and the blue line for the preferred verb condition.

Since the interaction effect of Verb Type ^*^ NP Predictability was also found at NP + 1 and NP + 2 (see Table 4), we conducted additional linear mixed-effects analyses to examine how the NP Predictability interacted with the Verb Type to affect the reading times at the two critical regions. Before the statistical analyses, we first visualized the data by plotting a distribution of NP cloze probabilities for each item in terms of different verb types, together with reading time data points (from NP + 1 region to facilitate our later discussion) (see Figure 2). We examined in which verb type the NP predictability may affect the reading time. All the data were subset into three groups in terms of Verb Type. The separate linear mixed-effects analyses were performed with these groups, with the log-transformed reading times as the dependent variable, NP Predictability as a fixed factor, and by-participants and by-items random slope for the main effects of NP Predictability. The statistical analyses were conducted on the data from each of the two critical regions (i.e., NP + 1, NP + 2) where the interaction effect of Verb Type ^*^ NP Predictability was found. The results are reported in Table 6.

**Table 6:** Reading time results affected by NP predictability with the data subset by verb type.

		***Estimate***	***Std. error***	***df***	***t***	***p***
AspV	NP + 1	−233.93	87.14	21.12	−2.684	0.014^*^
	NP + 2	−114.934	93.759	6.861	−1.226	0.261
PrefV	NP + 1	20.21	33.09	39.43	0.611	0.545
	NP + 2	6.998	31.121	580.291	0.225	0.822
NonPrefV	NP + 1	−22.98	83.32	28.01	−0.276	0.785
	NP + 2	−48.39	59.45	10.95	−0.814	0.433

*AspV, Aspectual Verb; PrefV, Preferred Verb; NonPrefV, Nonpreferred Verb*.

*** *p ≤ 0.001*;

** *p ≤ 0.01*;

* *p ≤ 0.05*;

*‘.'p ≤ 0.1*.

## Results

Mean reading times for all sentence types at the four critical regions are presented in Table 2, and illustrated in Figure 1. As presented in Table 4, the verb region and the NP region did not show the main effect of Verb Type and the interaction effect between Verb Type and NP Predictability; the effects were found at the NP + 1 and the NP + 2 regions.

Considering the main effect of Verb Type, at the NP + 1 region, the analyses revealed an effect of Verb Type [$\chi_{\left(2\right)}^{2}$ = 5.276, *p* = 0.071] (see Table 4). Aspectual verb sentences produced much longer reading time than Preferred verb sentences (42.74 ms) and Non-preferred verb sentences (33.55 ms), and the latter two did not yield a large difference in reading times (9.19 ms). Pairwise comparisons (see Table 5) revealed that the reading time differences reached the significance level both between the Aspectual verb condition and the Preferred verb condition (*Estimate* = −0.125, *SE* = 0.035, *t* = −3.560, *p* < 0.001), and between the Aspectual verb condition and the Non-preferred verb condition (*Estimate* = −0.057, *SE* = 0.029, *t* = −1.963, *p* = 0.050). Between the Preferred verb condition and the Non-preferred verb condition, there was a marginal difference found (*Estimate* = 0.069, *SE* = 0.037, *t* = 1.856, *p* = 0.064).

The NP + 2 region also illustrated the main effect of Verb Type [$\chi_{\left(2\right)}^{2}$ = 7.211, *p* = 0.027], exhibiting a similar pattern of reading times to the NP + 1 region. Compared with Aspectual verb sentences, both the Preferred verb and Non-preferred sentences were processed with shorter reading times (−25.23, −23.50 ms). Pairwise comparisons showed that there was a statistically significant difference of reading times between the Aspectual verb and Preferred verb sentences (*Estimate* = −0.095, *SE* = 0.031, *t* = −3.030, *p* = 0.002), and between the Aspectual verb and Non-preferred verb sentences (*Estimate* = −0.052, *SE* = 0.027, *t* = −1.955, *p* = 0.051), but not between the two types of non-coercion sentences (*Estimate* = 0.043, *SE* = 0.034, *t* = 1.285, *p* = 0.199).

Considering the interaction between Verb Type and NP Predictability, the significant effect was found at both the NP + 1 [$\chi_{\left(2\right)}^{2}$ = 10.54, *p* = 0.005] and the NP + 2 regions [$\chi_{\left(2\right)}^{2}$ = 5.881, *p* = 0.053] (see Table 4). The effect of NP Predictability on the reading time was only exhibited in the aspectual verb sentences (see Table 6), particularly at the NP+1 region (*Estimate* = −233.93, *SE* = 87.14, *t* = −2.684, *p* = 0.014). This effect was not observed in the other two sentence types at any region.

In sum, the main effects of Verb Type and the interaction between Verb Type and NP Predictability were found at the two post-critical regions, i.e., NP + 1 and NP + 2. As for the effect of Verb Type, it was mainly exhibited with the pairs of Aspectual verbs vs. Preferred verbs, and Aspectual verb vs. Non-preferred verb. As for the interaction between Verb Type and NP Predictability, the NP Predictability effect on the reading time was only demonstrated on the aspectual verb sentences at the NP + 1 region. The results seem to show that the NP predictability was at play, to some extent, in affecting the reading times of sentences in the aspectual condition. However, this was actually not true, and shall be discussed in the Discussion section. The main effect of Verb Type and the interaction of the two fixed effects did not emerge at the verb region and the NP region.

## Discussion

The current study investigates the processing of Mandarin sentences of complement coercion structure with aspectual verbs (i.e., aspectual verbs + entity-denoting noun). Three main findings were obtained: (1) At both the NP + 1 and the NP + 2 region, coercion sentences (i.e., with aspectual verbs) induced much longer reading time than the other two conditions of non-coercion sentences (i.e., with preferred and non-preferred verbs respectively), which reached the significance level. (2) At the critical NP region, no statistically significant difference in reading times was observed between the coercion condition and the two non-coercion conditions, although the former did elicit numerically longer reading time than the latter. (3) Within the two non-coercion conditions, there was no large difference found through all the regions of interest, although a marginal difference was observed at the NP + 1 region. The results are generally within our expectation and further discussed below.

The first finding indicates that interpreting sentences with complement coercion structure is more taxing and intensive than sentences with non-coercion structures. The result accords well with most of the previous psycholinguistic studies (e.g., McElree et al., 2001, 2006; Traxler et al., 2002, 2005; Pickering et al., 2005; Husband and Politzer-Ahles, 2016; Delogu et al., 2017; Xue and Liu, 2020). Sentences with complement coercion involves a type clash between the event-selecting verb and its entity-denoting complement noun. To resolve this clash in meaning, the language comprehension system is thought to implicitly shift the semantic type of the noun from an entity to an event to recover the implicit event information associated with the object argument (Pustejovsky, 1991, 1995; Jackendoff, 1997). This operation enriches the semantic composition of the structure (Jackendoff, 1997), which consequently requires more effort to process and thus triggers processing cost during real time comprehension (e.g., Pickering et al., 2005; Traxler et al., 2005; McElree et al., 2006; Frisson and McElree, 2008; Husband and Politzer-Ahles, 2016; Xue and Liu, 2020).

Alternatively, Delogu et al. (2017) attributed the elicited processing cost to the high surprisal of the relatively unpredictable entity argument following the event-selecting verb, and the coercion specific operations may influence later processing stages. In their eye-tracking study, they matched the predictability of the complement noun preceded by a coercing verb (e.g., *begin* *the book*) vs. a non-coercing verb (e.g., *buy* *the book*). However, although predictability was controlled to be almost alike, the coercion condition still generated reading time slowdowns than the non-coercion counterpart, in terms of total reading times at the object noun (but not “earlier” reading times). The result is quite consistent with what we have obtained here, and this, from our view, suggests that semantic enrichment goes beyond the surprisal to affect the interpretation of complement coercion expressions. In the present study, we incorporated the cloze probability of the target noun to the statistical models, and still found the significant effect of verb types. This indicates that the processing slowdown in the coercion noun is more likely driven by the type converting to recover the implicit event information, above and beyond the surprisal effect.

One may argue that the semantic predictability of the complement nouns, even though not the fundamental source of the reading time slowdowns, may play a role more or less in the on-line processing of complement coercion sentences. As presented in the Data Analysis section, our analyses revealed an interaction effect of the noun predictability and the verb type, which, particularly, was manifested at the NP + 1 word of the sentences with aspectual verbs (i.e., coercion) (see Table 6). However, taking a close look of this set of data, it was found that many of the predictability values of the nouns following the aspectual verbs were at 0% (see Figure 2), which manifested a floor effect (occurring when scores on a variable are approaching the possible lower limit) (Cramer and Howitt, 2004). To this point, it is hard to examine the effect of the predictability in the aspectual verb condition. The low predictabilities of the entity-denoting noun in this condition were within the expectation, since at this region an event/action-denoting complement is supposed to be there rather than the one denoting a physical object/entity. Interesting to note that the complement nouns following the non-preferred verbs were also relatively unexpected, similar to the ones following the aspectual verbs; however, there were still significant processing divergences detected between these two types of sentences. This further indicates that there is more likely an independent mechanism involved in the coercion sentences which gives rise to the demanding processing, most likely, the coercion operation according to the relevant literature.

The result that the processing cost is not detected at the critical noun region where the type-mismatch appears (but at the two subsequent regions) is not absolutely compatible with those self-paced results in Pickering et al. (2005), McElree et al. (2001), and Xue and Liu (2020), which reported either significant or marginal effect of verb type at the complement noun. In the Pickering et al. (2005), the processing difficulty arose only at the noun but not the post-noun region. In the McElree et al. (2001) study, the processing difficulty was generated at both the noun region and the post-noun region. Xue and Liu (2020) reported a marginal effect of condition at the noun and a significant effect of condition at the post-noun word. Note, however, that in the current study, although the reading times at the complement noun do not significantly differ between the aspectual coercion condition and the two non-coercion conditions, there are still numerical discrepancies displayed clearly: aspectual verb condition with 443.64 ms, preferred verb condition with 422.98 ms, and non-preferred verb condition with 425.54 ms. Furthermore, it is also necessary to note a salient difference in types of coercing verbs between the present study and the other three studies: in the current study, homogenous stimuli with only aspectual verbs (e.g., 开始 *kāi-shǐ* “begin/start”) were used; in the other three studies, however, heterogenous stimuli with aspectual verbs (e.g., *begin*), psychological verbs (e.g., *enjoy*), and other type-unspecified verbs (e.g., *try*) were included. The heterogenous stimuli issue was addressed in Katsika et al. (2012), Piñango and Deo (2016), and Lai (2017). Thus, we selected the homogenous stimuli to try to avoid any effect triggered by potential un-uniformed selection properties of different verb types. Given the spill-over effect elicited, it may be resulted from two possibilities. One is that coercion construction more likely requires interpolation of additional semantic structure to meet the constraint of the event-selecting verbs; the processing difficulty thus may occur soon after readers encounter the entity noun, as suggested in McElree et al. (2001) and Traxler et al. (2002). On the other hand, the delayed processing cost may be caused by the nature of the self-paced reading paradigm used. Even though the processing difficulty is triggered at the noun where the type mismatch occurs, since the task is acted with pressing buttons, the effect might be reflected after the noun. In particularly, the smaller variety of verbs used in the present study may have allowed participants to rush through the sentences more quickly, causing effects to appear after the critical word.

The third finding, regarding the marginal reading time discrepancy at the NP + 1 within the two non-coercion conditions (i.e., *preferred* and *non-preferred*) and the absence of this discrepancy at the NP + 2, indicates that the two types of expressions behave similarly during the real-time comprehension. The result differs from McElree et al. (2001), in which they reported reading time slowdowns at the noun of non-preferred expressions compared to preferred expressions. The more processing demands for the non-preferred expressions were suggested to be likely due to “the non-prototypical relationship” expressed between the verb and the object noun. For example, “writing a book” is a more prototypical activity for an author than “reading a book” as for the coercion expression *The author began the book*. But the potential reasons for this difference are not entirely clear. In the current study, the slightly more taxing processing detected on the NP + 1 words in the non-preferred sentences, however, disappear when readers encounter the word at the NP + 2 region, which, at least, indicates that the processing profile of the non-preferred sentences did not deviate largely from the processing profile of the preferred sentences. Our result largely aligns with Xue and Liu (2020), who did not find reading time discrepancy between non-preferred and preferred conditions either at the complement noun or the subsequent words. As pointed out by Traxler et al. (2002), in the non-coercion sentences, the verb specifies an activity and the default interpretation of the object noun is compatible with the verb's selectional restrictions; thus, there is no need of additional semantic processing at all in interpreting the sentences.

This study makes both theoretical and empirical contributions to the complement coercion phenomenon. It shows the presence of a processing cost of Mandarin sentences with complement coercion, particularly with aspectual verbs. The similar processing profiles exhibited in Mandarin–a language outside Indo-European languages family, compared with those in English and German, for instance, offer clear cross-linguistic evidence to the theory-building of complement coercion as a universal phenomenon. As mentioned previously, complement coercion has been studied extensively and systematically in Indo-European languages with both analytical analyses and empirical evidence. Our evidence on Mandarin Chinese extends the theoretical validity and applicability of complement coercion outside the Indo-European language family to show that syntactically-allowed semantic type shifting is cognitively more costly and engender difficulty in sentence processing. Moreover, the study makes a significant contribution to verify previous conflicting views on complement coercion in Mandarin Chinese. Although complement coercion has been postulated as existing in Mandarin by some scholars, there has never been direct empirical evidence. Given the suggested differences by Song (2015) between Mandarin and English, i.e., complement coercion is less pervasive in Mandarin with weaker/little potentials for some Mandarin event-selecting verbs to trigger coercion, it is doubtful if Mandarin sentences with complement coercion will evoke taxing processing. This study is determined to seek empirically valid evidence to verify previous claims. In addition, the current study is a follow-up of a preliminary study on the processing of Mandarin sentences with complement coercion configuration by Xue and Liu (2020) that looked at a wider range of potential coercing verbs, which, according to some recent studies (Katsika et al., 2012; Piñango and Deo, 2016), may be problematic given the distinct selectional properties of lexical items. Refining the experimental design applied in Xue and Liu (2020), the current study includes only a homogeneous verb type, i.e., aspectual verbs, which have been acknowledged as representatives of coercing verbs, in order to have a more rigid design and to obtain more convincing evidence for the processing patterns of Mandarin sentences.

A few limitations may need to keep in mind. Firstly, as mentioned, the cloze probability of the noun complement was not manipulated. Although this factor was included in our statistical models, it would be better if the variable could be controlled in the experimental design, such that the processing cost, observed on and/or after the target noun, could be exclusively attributed to the effect of verb type rather than other possible effects. Second, the study was carried out with the self-paced reading paradigm, which might not be the most natural way of examining the reading process compared with other on-line techniques, such as eye movements and event-related potentials (ERPs). To capture a comprehensive picture of complement coercion interpretation in Mandarin, further research may adopt other reliable methods to verify the findings reported here.

## Conclusion

The present study provides experimental evidence for processing costs in sentences involving complement coercion in Mandarin, especially with aspectual verbs combining with an entity-denoting complement. While the processing cost is not observed at the critical complement noun, it is detected at the regions directly following the noun. The absence of the processing cost at the critical region is attributed to the possibility that readers might have realized the type mismatch when encountering the entity object argument but more time is reflected at the later words to repair the mismatch, or the possibility that the nature of the self-paced reading paradigm applied in the current study may lead to spill-over effect. Overall, the results support the most of the previously relevant studies, and are consistent with the traditional semantic enrichment account for the processing difficulty. The study contributes plausible and compelling evidence to coercion studies cross-linguistically.

## Data Availability Statement

All experimental stimuli, data, as well as R-codes for analyses are available at https://osf.io/wd2g7/?view_only=9939497a78bd41d6a0ac01896ca61612.

## Ethics Statement

The studies involving human participants were reviewed and approved by Ethics Committee of the Department of Linguistics and Translation, City University of Hong Kong. The participants provided their written informed consent to participate in this study.

## Author Contributions

All authors contributed to the conception and design of the study, as well as manuscript revision. ML and SP-A supervised the project. WX performed the data collection and wrote the manuscript. WX and SP-A performed the statistical analyses. WX, ML, and SP-A revised the manuscript. All authors read the final manuscript and agreed to the submission of this version.

## Conflict of Interest

The authors declare that the research was conducted in the absence of any commercial or financial relationships that could be construed as a potential conflict of interest.

## Footnotes

^1^We thank a reviewer for the suggestion on an alternative method to evaluate multiple types of effects in a single model. The basic concept is to perform likelihood ratio tests in the way of comparing a maximal model to the same model but without the effect in question (see Piccinini, 2018). Note, however, that when using this method, we need to first make the categorical variable contrast coded (see Piccinini, 2016). By comparing the results obtained from the suggested method with the results obtained from our current method, both analyses reveal the main effect of Verb Type (particularly between Aspectual verb and Preferred verb conditions), which reaches the significance level at the words immediately after the NP complement. Differing from the current analyses, the suggested analyses reveal an effect of NP predictability at the NP + 1 region, which was not found from our current analyses. This effect, in our view, however, might not be a real effect to some extent, as discussed in the manuscript (see Discussion section for details). The suggested analyses also report two more complicated interaction effects between NP Predictability and *Contrasts* differences, i.e., NP Predictability: *Contrast 1* (between Verb Type 1 vs. Verb Type 2), and NP Predictability: *Contrast 2* (between Verb Type 1 vs. Verb Type 3). These two interaction effects are not directly relevant and useful to our questions on whether there is a clear effect of Verb Type yielded and whether there is a simple interaction effect between Verb Type and NP Predictability. Therefore, we reported the results obtained from the current method in the manuscript.
