# Supplementary material for: Processing of Complement Coercion With Aspectual Verbs in Mandarin Chinese: Evidence From a Self-Paced Reading Study
Source: Front Psychol. 2021 May 31;12:643571. doi: 10.3389/fpsyg.2021.643571 (PMC8201986; doi:10.3389/fpsyg.2021.643571)
Supplement: Supplementary file 1 [file Data_Sheet_1.DOCX]

Supplementary Material

# Experimental Stimuli

The 32 triplets of experimental stimuli are presented, with *Pinyin* (official romanization system for Mandarin), English glossary, and English translation. Each sentence of a triplet varies in the matrix verb only (separated by slashes). The first verb is the event-selecting aspectual verb, the second is the preferred verb, and the third is the non-preferred verb. The abbreviations involved are citied from Li and Thompson (1981) (CL = Classifier; CRS= Currently relevant state *le*; GEN = Genitive *de*; 3sg = third person singular pronoun).

(1) 医生开始/阅读/检查这份病例已经几个小时了。

*yī-shēng kāi-shǐ/yuè-dú/jiǎn-chá zhè-fèn bìng-lì yǐ-jīng jǐ-gè xiǎo-shí le*

doctor start/read/check this-CL medical-report already several hour CRS

‘The doctor has started/read/checked the medical report for several hous.’

(2) 艺术家继续/雕刻/塑造这个雕塑直到满意为止。

*yì-shù-jiā jì-xù/diāo-kè/sù-zào zhè-gè diāo-sù zhí-dào mǎn-yì wéi-zhǐ*

artist continue/engrave/shape this-CL sculpture until satisfied till

‘The artist continues/engraves/shapes the sculpture until he is satisfied with it.’

(3) 官员结束/阅读/审查这份报告之前需要请示领导。

*guān-yuán jié-shù/yuè-dú/shěn-chá zhè-fèn bào-gào zhī-qián xū-yào qǐng-shì lǐng-dǎo*

officer end/read/examine this-CL report before need request leader

‘The official needs to ask for instructions from higher authority before ending/reading/examining the written report.’

(4) 工匠完成/雕刻/修缮这个石碑之前喝了一杯茶水。

*gong-jiàng wán-chéng/diāo-kè/xiū-shàn zhè-gè shí-bēi zhī-qián hē-le yī-bēi chá-shuǐ*

craftsman finish/engrave/repair this-CL stele before drink one-CL tea

‘The craftsman drank a cup of tea before finishing/engraving/repairing the stele.’

(5) 警察停止/检查/拿起这个背包之后走向那个旅客。

*jǐng-chá tíng-zhǐ/jiǎn-chá/ná-qǐ zhè-gè bēi-bāo zhī-hòu zǒu-xiàng nà-gè lǚ-kè*

policeman stop/examine/pick up this-CL backpack after go-to that-CL tourist

‘The policeman went to the tourist after stopping/checking/picking up the backpack.’

(6) 园丁开始/修剪/维护这个花园需要投入很多时间。

*yuan-dīng kāi-shǐ/xiū-jiǎn/wéi-hù zhè-gè huā-yuán xū-yào tóu-rù hěn-duō shí-jiān*

gardener begin/trim/maintain this-CL garden need spend much time

‘The gardener needs to spend much time in starting/trimming/maintaining the garden.’

(7) 证人完成/提供/证实这份证词就会洗刷多年冤屈。

*zhèng-rén wán-chéng/tí-gōng/zhèng-shí zhè-fèn zhèng-cí jiù-huì xǐ-shuā duō-nián yuan-qū*

witness finish/provide/affirm this-CL testimony then right many-year injustice

‘It will help the witness to right the injustice after finishing/providing/affirming the testimony’.

(8) 老师停止/朗读/解释这篇课文并且快速走上讲台。

*lǎo-shī tíng-zhǐ/lǎng-dú/jiě-shì zhè-piān kè-wén bìng-qiě kuài-sù zǒu-shàng jiǎng-tái*

teacher stop/read/explain this-CL article and quickly go-up platform

‘The teacher stopped/read/explained the article and then go up quickly onto the platform.’

(9) 顾客开始/填写/查看这份问卷之前看过身份证。

*gù-kè kāi-shǐ/tián-xiě/chá-kàn zhè-fèn wèn-juàn zhī-qián kàn-guò shēn-fèn-zhèng*

customer start/fill-in/check this-CL questionnaire before look-at ID-card

‘The customer took a look at the ID card before starting/filling in/ checking the questionnaire.’

(10) 教授完成/发表/指导这篇论文我们感到特别高兴。

*jiào-shòu wán-chéng/fā-biǎo/zhǐ-dǎo zhè-piān lùn-wén wǒ-men gǎn-dào tè-bié gāo-xìng*

professor finish/publish/instruct this-CL essay we feel very happy

‘We are excited that the professor has finished/published/supervised the paper.’

(11) 乐队停止/演奏/排练这类歌曲打算尝试新的挑战。

*yuè-duì tíng-zhǐ/yǎn-zòu/pái-liàn zhè-lèi gē-qǔ dǎ-suàn cháng-shì xīn-de tiǎo-zhàn*

band stop/play/rehearse this-CL song plan try new challenge

‘The band stopped/played/rehearsed this kind of songs and planned to try more challenging ones.’

(12) 掌柜继续/管理/装修这个店铺需要投入大量资金。

*zhǎng-guì jì-xù/guǎn-lǐ/zhuāng-xiū zhè-gè diàn-pù xū-yào tóu-rù dà-liàng zī-jīn*

shopkeeper continue/manage/renovate this-CL shop need spend much money

‘It will take much money for the shopkeeper to continue/manage/renovate the shop.’

(13) 导演停止/拍摄/认可这个广告可能因为代言人吧。

*dǎo-yǎn tíng-zhǐ/pāi-shè/rèn-kě zhè-gè guǎng-gào kě-néng yīn-wèi dài-yán-rén ba*

director stop/film/approve this-CL commercial maybe because spokesman

‘The director stopped/filmed/approved the commercial probably due to the spokeman.’

(14) 建筑师完成/设计/修建这栋大楼是在合约期限内。

*jiàn-zhù-shī wán-chéng/shè-jì/xiū-jiàn zhè-dòng dà-lóu shì-zài hé-yuē qī-xiàn nèi*

architect finish/design/build this-CL building is contract term within

‘The architect finished/designed/built the building within the contract term.’

(15) 模特继续/保持/模仿这个姿势已经几个小时了。

*mó-tè jì-xù/bǎo-chí/mó-fǎng zhè-gè zī-shì yǐ-jīng jǐ-gè xiǎo-shí le*

model continue/keep/imitate this-CL posture already several hour CRS

‘The model has continued/kept/imitated the posture for several hours.’

(16) 观众停止/观看/批评这部电影因为演员都是新人。

*guān-zhòng tíng-zhǐ /guān-kàn /pī-píng zhè-bù diàn-yǐng yīn-wèi yǎn-yuán dōu-shì xīn-rén*

audience stop/watch/criticize this-CL movie because actor is junior

‘The audience stopped/watched/criticized the movie in that all the actors were junior.’

(17) 农民开始/开垦/购买那片荒地已经得到政府批准。

*nóng-mín kāi-shǐ/kāi-kěn/gòu-mǎi nà-piàn huāng-dì yǐ-jīng dé-dào zhèng-fǔ pī-zhǔn*

farmer start/reclaim/buy that-CL wasteland already obtain government approval

‘The farmer has received the government’s approval of starting/reclaiming/buying the wasteland.’

(18) 导演停止/指导/策划这部电影引起观众强烈不满。

*dǎo-yǎn tíng-zhǐ/zhǐ-dǎo/cè-huà zhè-bù diàn-yǐng yǐn-qǐ guān-zhòng qiáng-liè bù-mǎn*

director stop/instruct/plan this-CL movie make audience opposition

‘The director stopped/instructed/planned the movie, which caused the opposition of the public.’

(19) 木匠完成/建造/修补这栋房子大约是在去年冬天。

*mù-jiàng wán-chéng/jiàn-zào/xiū-bǔ zhè-dòng fang-zi dà-yuē shì-zài qù-nián dōng-tiān*

carpenter finish/build/renovate this-CL building around is last-year winter

‘The carpenter finished/built/renovated this building in the winter of last year.’

(20) 学生继续/阅读/赏析这篇文章直到老师打断他们。

*xué-shēng jì-xù/yuè-dú/shǎng-xī zhè-piān wén-zhāng zhí-dào lǎo-shī dǎ-duàn tā-men*

student continue/read/analyze this-CL article until teacher interrupt them

‘The students continued/read/analyze the article until their teacher interrupted them.’

(21) 程序员结束/编写/修改这些代码之后倒头大睡。

*chéng-xù-yuán jié-shù/biān-xiě/xiū-gǎi zhè-xiē dài-mǎ zhī-hòu dǎo-tóu-dà-shuì*

programmer end/write/revise this-CL code after sleep

‘The programmer went to sleep after ending/writing/revising the codes.’

(22) 歌唱家停止/演唱/批评这首歌曲是在专辑出版前。

*gē-chàng-jiā tíng-zhǐ/yǎn-chàng/pī-píng zhè-shǒu gē-qǔ shì-zài zhuān-jí chū-bǎn qián*

singer stop/sing/criticize this- CL song is album public before

‘The singer stopped/sang/criticized the song before the publish of the album.’

(23) 保洁员开始/打扫/进入这个房间大约是在早饭前。

*bǎo-jié-yuán kāi-shǐ/dǎ-sǎo/jìn-rù zhè-gè fang-jiān dà-yuē shì-zài zǎo-fàn qián*

cleaner start/clean/enter this-CL room around is breakfast before

‘The cleaner started/cleaned/entered the room before the breakfast.’

(24) 游客完成/填写/领取这张表格就能合法入境旅游。

*yóu-kè wán-chéng/tián-xiě/lǐng-qǔ zhè-zhāng biǎo-gé jiù-néng hé-fǎ rù-jìng lǚ-yóu*

tourist finish/fill-in/get this-CL form then legal enter tourism

‘The tourists received the lawful admission of the land after finishing/filling in/ getting the form.’

(25) 表妹结束/阅读/拥有这本小说是在昨天午睡前。

*biǎo-mèi jié-shù/yuè-dú/yōng-yǒu zhè-běn xiǎo-shuō shì-zài zuó-tiān wǔ-shuì qián*

cousin end/read/have this-CL novel is yesterday nap before

‘The cousin ended/read/got the novel before taking a nap yesterday.’

(26) 裁缝继续/裁剪/修改这件衣服期间一直没有休息。

*cái-féng jì-xù/cái-jiǎn/xiū-gǎi zhè-jiàn yī-fú qī-jiān yī-zhí méi-yǒu xiū-xī*

tailor continue/cut/alterate/this-CL clothes before keep no rest

‘The tailor did not take a rest during the time she continued/cut/alterated the clothes.’

(27) 消费者停止/购买/认可这个产品可能因为生产地。

*xiāo-fèi-zhě tíng-zhǐ/gòu-mǎi/rèn-kě zhè-gè chǎn-pǐn kě-néng yīn-wèi sheng-chǎn-dì*

consumers stop/buy/satisfy this-CL product probably because place-of-origin

‘The consumers stopped/bought/satisfied with the product probably due to the place of origin.’

(28) 科学家开始/研究/主持这个项目遭到大家反对。

*kē-xué-jiā kāi-shǐ/yán-jiū/zhǔ-chí zhè-gè xiàng-mù zāo-dào dà-jiā fǎn-duì*

scientist begin/investigate/host this-CL project incur people opposition

‘Scientists began/investigated/hosted this project, which incurred the opposition.’

(29) 糕点师完成/制作/购买这个蛋糕之后就去喝酒了。

*gāo-diǎn-shī wánc-héng/zhì-zuò/gòu-mǎi zhè-gè dàn-gāo zhī-hòu jiù-qù hēj-iǔ CRS*

baker finish/make/buy this-CL cake after go drink beer

‘The baker went to drink beer after finishing/making/buying the cake.’

(30) 船长停止/驾驶/指挥这艘轮船因为这是他的职责。

*chuán-zhǎng tíng-zhǐ/jià-shǐ/zhǐ-huī zhè-sōu lún-chuán yīn-wèi zhè-shì tā-de zhí-zé*

captain stop/drive/command this-CL ship because this-is 3sg-GEN duty

‘It is the captain’s duty to stop/drive/command the ship.’

(31) 钢琴家继续/演奏/谱写这首曲子为了展示其才艺。

*gang-qín-jiā jì-xù/yǎn-zòu/pǔ-xiě zhè-shǒu qǔ-zi wèi-le zhǎn-shì qí cái-yì*

pianist continue/perform/write this-CL song for show the talent

‘The pianist continued/performed/wrote the song to show his talent.’

(32) 厨师结束/制作/欣赏这道美食之后就去喝酒了。

*chú-shī jié-shù/zhì-zuò/xīn-shǎng zhè-dào měi-shí zhī-hòu jiù-qù hē-jiǔ CRS*

chef end/make/enjoy this-CL delicious-food after go drink-beer CRS

‘The chef went to drink beer after ending/making/enjoying the meal.’
